# KRAB-Zinc Finger Proteins: A Repressor Family Displaying Multiple Biological Functions

**DOI:** 10.2174/13892029113149990002

**Published:** 2013-06

**Authors:** Angelo Lupo, Elena Cesaro, Giorgia Montano, Diana Zurlo, Paola Izzo, Paola Costanzo

**Affiliations:** 1 Dipartimento di Medicina Molecolare e Biotecnologie Mediche, Università di Napoli “Federico II”, Via S. Pansini 5, 80131 Napoli, Italy;; 2 Dipartimento di Scienze per la Biologia, la Geologia e l’Ambiente, Facoltà di Scienze, Università del Sannio, Via Port’Arsa 11, 82100 Benevento, Italy

**Keywords:** Apoptosis, Evolution, KAP-1 corepressor, KRAB domain, Metabolism, Transcriptional repression, Zinc finger.

## Abstract

Zinc finger proteins containing the Kruppel associated box (KRAB-ZFPs) constitute the largest individual family of transcriptional repressors encoded by the genomes of higher organisms. KRAB domain, positioned at the NH2 terminus of the KRAB-ZFPs, interacts with a scaffold protein, KAP-1, which is able to recruit various transcriptional factors causing repression of genes to which KRAB ZFPs bind. The relevance of such repression is reflected in the large number of the KRAB zinc finger protein genes in the human genome. However, in spite of their numerical abundance little is currently known about the gene targets and the physiological functions of KRAB- ZFPs. However, emerging evidence links the transcriptional repression mediated by the KRAB-ZFPs to cell proliferation, differentiation, apoptosis and cancer. Moreover, the fact that KRAB containing proteins are vertebrate-specific suggests that they have evolved recently, and that their key roles lie in some aspects of vertebrate development. In this review, we will briefly discuss some regulatory functions of the KRAB-ZFPs in different physiological and pathological states, thus contributing to better understand their biological roles.

## INTRODUCTION

Transcriptional control in eukaryotic cells is an extremely complex process involving a large number of transcription factors (TFs) and cofactors that regulate the assembly of transcription-initiation complexes and the rate at which transcription is initiated. Additionally, a variety of enzymes modulate chromatin structure via changes in DNA methylation, histone modifications and nucleosome positioning. 

An essential feature in the functioning of TFs is the presence of specific DNA-binding motifs that facilitate the binding of TFs to the promoters of target genes. The interactions between TFs and their DNA-binding sites are part of the gene regulatory networks that control development, core cellular processes and responses to environmental perturbations.

One of the most abundant DNA-binding motifs in eukaryotic TFs is the zinc finger (ZF). Different classes of zinc finger domains have been identified and characterized according to the nature and spacing of their zinc-chelating residues [1]. The canonical ZF motif, C2H2 (also referred to as Kruppel-like), comprises 28 to 30 amino acid residues and its structure is stabilized by a zinc ion coordinated by four highly conserved residues, two cysteines and two histidines [2]. The stably folded structure consists of two to three beta strands and one alpha helix. The alpha helix mediates DNA binding through non-covalent interactions between three of its amino acid residues and three adjacent bases within the DNA major groove [3]. 

C2H2 zinc finger proteins contain from 1 to more than 30 ZF motifs and represent the largest family of regulatory proteins in mammals, involved in transcriptional regulation through the binding of their sequence-specific DNA binding motifs to the promoter regions of the genes. In fact, genes encoding C2H2 zinc finger proteins, together with olfactory receptor genes, represent more than 2% of the human genome [4, 5]. However, variations in the amino acid sequence of the finger domains and spacing, as well as in zinc finger number and higher-order structure, may increase the ability of these proteins to bind multiple different ligands such as RNA, DNA-RNA hybrids and even proteins, thus highlighting the structural and functional versatility of this protein family [6, 7]. Moreover, as well as binding to DNA, a number of C2H2 zinc finger proteins can also bind to RNA, thereby creating an additional level of gene regulation by connecting transcriptional and post-transcriptional regulatory processes [8, 9].

In addition to containing variable numbers of multifunctional ZF domains, most C2H2 zinc finger proteins contain other conserved domains that contribute to the protein functions. These include the BTB/POZ domain (Broad-Complex, Tramtrack, and Bric-a-brac/poxvirus and zinc finger), the SCAN domain ((SRE-ZBP, CTfin51, AW-1 and Number18 cDNA) and the KRAB domain (Kruppel-Associated Box) [10-12].

The KRAB domain is a potent transcriptional repression module and is located in the amino-terminal sequence of most C2H2 zinc finger proteins [13, 14]. It binds to corepressor proteins and/or transcription factors via protein-protein interactions, causing transcriptional repression of genes to which KRAB zinc finger proteins (KRAB-ZFPs) bind [15]. In humans, KRAB-ZPFs represent approximately one third of about 800 different zinc finger proteins and constitute one of the largest family of transcriptional regulators. In fact, due to the presence of the KRAB domain, a powerful transcriptional repressor domain, most members of the KRAB-ZFPs family have a role in regulating embryonic development, cell differentiation, cell proliferation, apoptosis, neoplastic transformation and cell cycle regulation [16]. 

There are essentially three major ways through which repressor proteins can down-regulate specific genes: inhibition of the basal transcription machinery, ablation of activator function and remodelling/compaction of chromatin. The first two mechanisms result from the repressor’s direct contact with RNA polymerase or a component of the basal transcription complex and activator, respectively; these repression mechanisms have long been studied in prokaryotes. The third mechanism was discovered more recently and requires the presence of additional proteins that affect chromosomal structure [17]. KRAB-ZFPs represent such an example of repressor proteins that obtain gene-specific transcriptional repression by interacting with chromatin-remodelling factors. In this review, we will attempt to describe the structural and functional features of some KRAB-ZFPs and in doing so, we will focus on the multiple functions that these proteins have.

## EVOLUTION AND STRUCTURE OF KRAB ZINC FINGER PROTEINS

The KRAB-ZFP gene family represents a more recent evolutionary product and its expansion in the genome of vertebrate tetrapods could indicate the acquisition of new functions to sustain differentiation and speciation. Despite the huge number of members belonging to this group of genes, KRAB-ZFP genes and their transcriptional targets are still far from being fully understood. Investigating the molecular mechanisms that caused the generation of this gene family during species evolution is crucial in the understanding of their functions. 

A comparative analysis of mammalian genomes revealed the existence of a large and highly conserved number of genes that originated through repeated cycles of duplications from a single ancestral gene. After their duplications, these new genes diversified their coding regions to produce novel proteins with new biological functions. Interestingly, these genes have been found clustered at particular sites on chromosomes suggesting the existence of a common repertoire of regulatory sequences and a coordinated mechanism of their gene expression [18-20]. In the mammalian genome, the gene families encoding olfactory receptors (OLFR), a-globins, KYR proteins and KRAB-ZFPs are the most representative within this class of clustered genes [21-24]. Surprisingly, consistent data from several laboratories demonstrate that, unlike the other aforementioned gene families, only the genes encoding KRAB-ZFPs are differentially expressed in various tissues during differentiation and development, indicating that these genes have functions unique to mammalian evolution and, specifically, molecular processes that establish the phenotypic differences between vertebrates and other species [10, 11, 16, 25]. Therefore, expression of KRAB-ZFP genes is independent of their genomic localization, as well as of nearby paralogues generated through gene duplications within the same gene cluster. These paralogues, as new members of the KRAB-ZFP family, show different expression patterns and novel non-redundant functions. It is very interesting that the expression of several target genes, which are crucial in different pathways, could be driven by a similar molecular mechanism involving KRAB-ZFP [16].

In the human genome, 742 structurally different KRAB-ZFPs, generated from 423 genes, have been identified to date. Within this group of genes, 384 are clustered at specific loci and most of them reside in 25 major clusters. One of the most extended gene clusters, which has been proved to be affected by duplications and subsequent purifying selection, is located on human chrosomome 19 (HSA19) [21]. This gene cluster has long been considered unique in primates and, for this reason, has been extensively investigated. Chrosomome 19 includes more than 200 KRAB-ZFP gene loci (of the above cited 423) clustered at 11 sites and about one hundred of them are present as functional orthologues in primate, canine and rodent lineages [18]. Comparison between homologous clusters from human chromosome 19 (HSA19q13.2) and mouse chromosome 7 (Mmu7) revealed the existence of 21 human and 10 mouse genes that were closely related. More interestingly, within these individual homologous clusters, one mouse gene was related to 10 homologous human genes, while one human gene was evolutionarily related to 6 mouse genes [18]. These observations indicated that both clusters originated from common ancestral genes and that duplication events and loss of related members occurred independently in the primate and rodent lineages after divergence, producing additional new members of KRAB-ZFPs in humans and a reduced number of KRAB-ZFPs in mice [18]. Another intriguing feature derived from the comparison between the species was that genes generated through duplications were located within the same cluster in the same order in humans and mice. Moreover, these genes were closely related to their parents, and showed a related sequence and exon/intron organization [21]. Although additional information could come out from the comparison between species in the search for specific orthologues, the facts that only 103 of the 423 human KRAB-ZFP genes are orthologous to their counterparts in the mouse genome and that about 200 genes represent primate-specific genes suggest that this latter class of genes confers an evolutionary advantage to vertebrate tetrapods [21]. 

Further investigations on another mammalian KRAB-ZFP cluster located at HSA5q35.3 and more recent studies on the human cluster located on chromosome 8 at position 8q24.3 indicate a strong conservation of the KRAB and zinc finger domains of KRAB-ZFPs in mammalian species (humans, chimps, dogs and mice). Moreover, these conserved clusters contain new members of the KRAB-ZFP gene family resulting from a lineage-specific expansion in humans that gave new genes with specialized functions. A simultaneous loss or partial deletion of these functional domains, on the contrary, confirm that ongoing evolution has provided fewer orthologues of the KRAB-ZFP family in rodents [21, 25]. 

**Fig. (1A):**
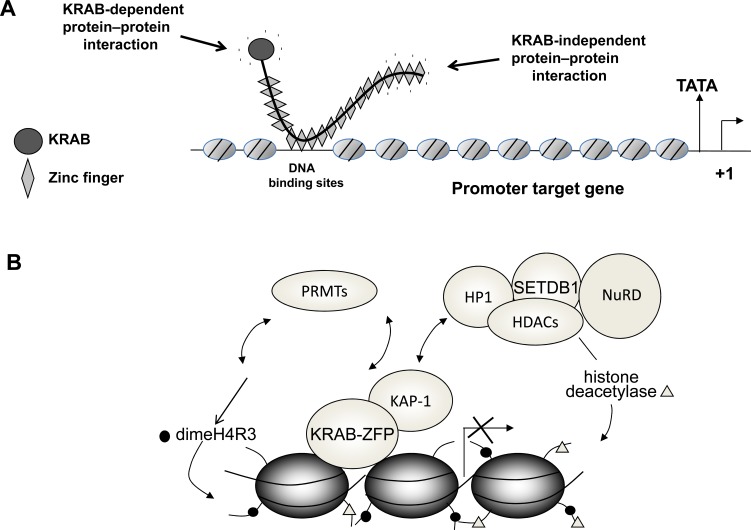
Zinc finger-mediated binding of a typical KRAB-ZFP to the promoter target gene. (**B**) Recruitment to chromatin of co-regulators and ATP-dependent activities that modify chromatin (PRMTs,HDACs, SETDB1, NuRD) mediated by KRAB/KAP-1 complex.

The functional role of KRAB-ZFPs depends on the organization of the different protein modules, i.e., the KRAB repressor domain located at the NH2 terminus and the DNA-binding domains present at the COOH terminus. The KRAB domain, mediating protein-protein interactions, binds to corepressor proteins and/or transcription factors, causing transcriptional repression of the genes to which KRAB-ZFPs bind (Fig. **1A** and **B**). Indeed, this domain usually consists of an A box and a B box. The A box plays a key role in repression by binding to corepressors, while the B box enhances the repression mediated by the A box through unknown mechanisms [10].

Three closely related subfamilies of KRAB-ZFPs have been found: one carrying the A box only, another having both the A and B boxes, and the third carrying the A box and a divergent “b” box [16]. Since these protein domains are generated through translation of the separate exons, it is coincevable that this modular design of KRAB-ZFP genes may foster the acquisition of new functions. 

Most human KRAB-ZFP genes include both the KRAB-A and KRAB-B domains in their sequences, whereas in mice, the KRAB-A domain is conserved and the KRAB-B is almost always deleted [18]. Less common regulatory motifs, KRAB-b, KRAB-BL and KRAB-C, have also been observed in KRAB-ZFPs [21]. 

Besides the KRAB domain, the SCAN (SRE-ZBP, CTfin51, AW-1 and Number18 cDNA) and the BTB (Broad complex Tramtrack Bric-a-brac) domains have been found at the NH2 terminus of KRAB-ZFPs [11, 12]. While the SCAN domain is involved in mediating protein-protein interactions, the BTB motif acts as a dimerisation domain. SCAN and KRAB domains are vertebrate-specific, whereas the BTB motif is also present in insects. The KRAB repression module is specific to the genomes of vertebrate tetrapods. It is absent in the zinc finger proteins derived from fish,* Drosophila*, plants, fungi and yeast, but present in frog, chicken, rat, mouse and human genomes [18]. This expression profile suggests that the KRAB domain is a rather recent product of evolution and that the expansion of the KRAB-ZFP family in vertebrate tetrapods is due to new functions related to differentiation and speciation [18]. 

Mammalian KRAB-ZFPs usually have a very high number of zinc finger domains located at the COOH terminus; they contain on average 12 domains, but in some proteins, 30 or more zinc finger domains have been found [19, 26]. KRAB-ZFPs anchor themselves to the canonical DNA-binding sites on the promoter of the target genes through two or three zinc finger domains, using the remaining domains to catch several different proteins through protein-protein interactions [26]. In mammalian catalogues of KRAB-ZFPs, frequent variations have been observed in the sequences as well as the number of zinc finger domains. Comparison between homologous clusters from human chromosome 19 (HAS 19q13.2) and mouse chromosome 7 (Mmu7) confirmed that human KRAB-ZFPs differed from their mouse counterparts in both the number of zinc finger domains and the sequences, thus suggesting that the divergence between the species might have resulted from a dramatic difference in DNA-binding properties [27]. This functional divergence between the two species could explain the difference in gene expression because variations in the structures of regulatory elements, through duplications, amplifications or deletions of the zinc finger domains, contribute to functional diversification.

## MOLECULAR MECHANISMS OF KRAB-ZFP-MEDIATED TRANSCRIPTIONAL REGULATION

Transcriptional repression mediated by KRAB-ZFPs requires interaction with chromatin-remodelling factors. Indeed, KRAB-ZFPs bind to the corresponding DNA sequence through their zinc finger domain, while the KRAB domain interacts with the corepressor protein, KAP-1 (KRAB-associated protein 1). The N-terminus of KAP-1 contains an RBCC (Ring finger/B box/Coiled-Coil) domain that binds the KRAB module as a homotrimer. The central region of KAP-1 includes a hydrophobic pentapeptide that interacts with the chromoshadow domain of heterochromatin protein 1 (HP1). The C-terminus tandem Plant homeodomain (PHD) and bromodomain of KAP-1 act as scaffold domains that recruit histone deacetylases and chromatin remodeling activities (such as NuRD complex), histone lysine-methyl transferase (such as SETDB1) to the promoters of target genes, thus initiating ATP-dependent activities that modify the chromatin. This results in silenced gene expression by enhancing the facultative heterochromatin as shown in (Fig. **1B**) [28-33]. 

Due to the limited number of KRAB-ZFP target genes identified so far, the transcriptional repressor complex has been mainly studied using artificial assays. While it is well known that KRAB-ZFPs strongly repress transcription *in vitro*, little is known about their role and the molecular mechanism of transcriptional repression* in vivo.*

Novel insights into chromatin modifications that are required for* in vivo* KRAB-ZFP repression of gene transcription arose from our studies on ZNF224, the human aldolase A gene repressor [34]. We showed that PRMT5, a type II protein arginine methyltransferase, played a crucial role in ZNF224-mediated transcriptional repression by methylating arginine 3 in histone H4. This indicated that PRMT5 may be another key mediator in the regulation of KRAB-ZFP-mediated repression [35] (Fig. **1B**).

Recently, genome-wide studies of KRAB–ZFP and KAP1 DNA-binding patterns have been performed in an attempt to propose new models of transcriptional repression* in vivo.* Groner *et al.* reported that KRAB/KAP1 recruitment induced long-range repression through the spread of heterochromatin in humans. Indeed, they suggested that KRAB–mediated repression was established by the long-range spreading of repressive chromatin marks, such as H3K9me3 and heterochromatin protein 1 (HP1), between the repressor binding site and the promoter that can be located several tens of kilobases away from the repressor’s primary docking site [36]. Therefore, the authors speculated that the dysregulation of KRAB/KAP1-mediated epigenetic changes could be involved in the long-range epigenetic silencing of large chromosomal regions observed in cancer cells. Furthermore, the same authors recently studied the impact of specific genomic features on KRAB/KAP1-induced silencing, suggesting that the genes most susceptible to KRAB/KAP1-induced silencing were in genomic regions of high gene activity and that pre-deposition of repressive histone marks to a gene increased its susceptibility to KRAB/KAP1-mediated repression [37].

KRAB–ZFPs are also involved in the generation of site-specific DNA methylation patterns during early embryogenesis; thus, these transcription factors contribute to the genome-wide establishment of epigenetic marks that are maintained during development [38]. This function was well studied for the ZFP57; in fact, Queneville *et al.* [39]. demonstrated that in embryonic stem cells, the selective ZFP57/KAP1 binding to a methylated hexanucleotide takes part in the maintenance of asymmetric histone modifications, heterochromatinization, and DNA methylation of Imprinting Control Regions (ICR). Moreover, the structure of the DNA binding motif of ZFP57 has been determined [40] and mutations of ZFP57 associated to Transient neonatal diabetes mellitus 1 (TNDM1) have been demonstrated to affect DNA binding activity [41].

Other intriguing studies conducted by Farnham’s group [42, 43] raised some doubts about the currently accepted model of KAP-1 recruitment in the human genome. They showed that at least two mechanisms for recruiting KAP1 may exist, one involving KRAB-ZFPs and another involving other DNA-binding proteins not yet identified. When KAP1 is recruited by KRAB-ZFPs, a striking feature is its enrichment at the 3’ends of the KRAB-ZNF genes. Recruitment to promoter regions could be mediated by a novel mechanism independent of the KRAB-ZPF. The authors of these studies suggested that the function of the KAP1/heterochromatin complex at the 3’ends of KRAB-ZFP genes is to deposit H3K9me3 and heterochromatin protein 1, and thus, maintain a heterochromatic state that reduces recombination-mediated deletion at KRAB-ZFP gene clusters. 

The ZBRK1 (Zinc finger and BRCA1-interacting protein with KRAB domain-1) protein is a typical member of the KRAB-ZFP family, which contains a KRAB domain at the NH2 terminus and a C-terminal repression domain (CTRD) at the COOH terminus. The CTRD domain binds the corepressor BRCA1, which recruits histone deacetylase complexes to the promoter of specific genes to repress transcription. The N-terminal KRAB domain of ZBRK1 may act in a BRCA1-independent manner by recruiting the corepressor KAP1 [44, 45]. Although the molecular mechanism of ZBRK1 transcriptional repression needs to be further investigated, it is tempting to speculate that through its partner proteins such as BRCA1 and KAP1, ZBRK1 might differentially regulate the expression of a broad spectrum of promoters. The eight C2H2 zinc fingers of ZBRK1 have two roles, recognizing a specific DNA-binding element and being involved in protein interactions. 

The zinc finger protein Nizp1 belongs to the SCAN-KRAB subfamily of C2H2-type zinc finger proteins that have a complete KRAB repression domain or, more frequently, only the A domain [12]. Nizp1 contains an N-terminal SCAN box associated with a KRAB A domain and a C-terminal C2HR motif followed by four classical C2H2-type zinc fingers that are capable of binding to DNA directly. The C2HR motif mediates protein-protein interaction with the NSD1 histone lysine methyltransferase and can repress transcription in an NSD1-dependent and KAP1-independent manner. The KRAB domain of Nizp1 seems to support C2HR domain-mediated transcriptional repression, in addition to its own role in transcriptional repression [46]. Another recent study identified the Nizp1 KRAB domain as a possible transcriptional activation domain [47]. Indeed, although known as transcriptional repressors based on *in vitro* analysis, recent findings indicate that some KRAB-ZFP can activate gene transcription *in vivo*. For example, the protein ZNF480 was described as a positive regulator in MAPK-mediated signalling pathways, leading to the activation of AP-1 and SRE [48]. Moreover, ZBRK1 can play a dual role in gene regulation by interacting with different co-regulators. In fact, while BRCA1 is a corepressor of ZBRK1 [44], Ataxin-2 has been demonstrated to be a coactivator of ZBRK1, interacting with ZBRK1 to activate its own transcription [49]. Furthermore, overexpression of ZBRK1 in Hela or U2OS cells increases the expression of numerous genes [50], thus supporting its role as a transcriptional activator, although the molecular mechanism involved is still largely unknown. 

ZNF263 is a C2H2 protein that contains 9 zinc finger domains, a KRAB repression domain and a SCAN domain. By a genome-wide ChIP-sequencing approach, more than 5000 binding sites for ZNF263 were identified in K562 cells and many of the ZNF263 target genes were found to be positively regulated by ZNF263 [51], further supporting the idea that KRAB-ZFPs can have both positive and negative effects on transcription.

Finally, another characteristic of Kruppel zinc finger motifs is their ability to not only bind DNA, but also RNA. There are some examples of KRAB-ZFP implicated in both transcriptional regulation and RNA processing. Among these, ZNF74 and ZNF224 have been shown to exist as multiple protein isoforms generated through alternative promoter usage and splicing. Both ZNF74 and ZNF224 isoforms exhibit differences in their transcriptional repression abilities and nuclear partitioning, with the isoforms devoid of the KRAB box being found in splicing speckle domains and able to bind RNA [52-54]. By contrast, the ZNF74 and ZNF224 isoforms containing the full KRAB A and B boxes exhibit strong transcriptional repression and localize in a diffuse pattern throughout the nucleus. These findings indicate that different KRAB-ZFPs may perform different functions, being involved in RNA processing and transcriptional regulation. It is also possible to speculate that alternative splicing may be a common means of modulating the multifunctionality of these zinc finger proteins.

## KRAB ZINC FINGER PROTEINS AND METABOLISM

KRAB-ZFP-mediated transcriptional repression controls the expression of a wide category of genes in different tissues during development and differentiation, as well as in response to environmental changes like nutrition, fasting and hormone stimulation. It is usually accepted that metabolism is regulated through three types of molecular mechanisms. First, a metabolic pathway can be modulated through the allosteric mechanism that controls activity of a regulatory enzyme via a conformational change of its structure. The rate of a metabolic pathway is also regulated by covalent modifications of the structure of the key enzyme. Phosphorylation, acetylation, glycosylation, sumoylation and methylation are the most common reactions that modify the structure of an enzyme, inducing a shift in the equilibrium between the active and inactive form of the enzyme to stimulate or repress its activity. Both these mechanisms result in a rapid and extremely efficient mode of regulating the rate of metabolic pathways. The third mechanism, which requires more time to achieve an increase (or decrease) in the rate and efficiency of a metabolic pathway, is the transcriptional control. Transcription factors can activate or inhibit groups of target genes in response to stimulation by nutritional or hormonal signals. Significant experimental data on KRAB-ZFPs that regulate the genes involved in metabolism have been accumulated and are now available. We report below some examples of KRAB-ZFPs that are involved in the transcriptional regulation of some pathways related to oxidative metabolism. 

ZNF202 is a KRAB-ZFP that binds to the regulatory elements present in the promoter regions of genes involved in lipid metabolism [55]. The target genes of ZNF202 can be distinguished into three groups. Firstly, ZNF202 regulates the transcription of genes encoding the structural components of lipoproteins like apoAIV, apoCIII, apoE and apoA1 [56-58]. Secondly, ZNF202 modulates the transcription of genes involved in lipid processing like those encoding LPL, lecithin-cholesterol acyltransferase and hepatic triglyceride lipase [56]. The functions of the genes included in these two classes are crucial for controlling homeostasis of lipids and cholesterol. The third group of ZNF202-dependent genes includes some (HNF4, insulinoma-associated gene 1, beta-3 adrenergic receptor and VEGF) that participate in particular metabolic processes [55, 56]. For example, beta-3 adrenergic receptor variants have been associated with body mass index, insulin resistance and plasma lipid levels [59-61]. Transcriptional control of the genes involved in the regulation of lipid metabolism is provided by the binding of ZNF202 to specific sequences on the promoters of target genes. This consensus sequence is a G-rich motif (5’-GGGGT-3’) [55]. A convincing indication for the role of ZNF202 in repression came out from the observation that upregulation of target genes is accompanied by a downregulation of ZNF202 expression [57]. It is interesting to note that ZNF202, besides the KRAB domain, has an additional SCAN domain at the NH2-terminus. The presence of this domain prevents the binding of KAP1 to ZNF202, thereby removing its repression ability and mediating interactions with coactivators [55]. The SCAN domain can therefore confer ZNF202 a specific ability to bind other protein partners through protein-protein interactions, shifting its role from repression to activation. 

Other KRAB-ZFPs that regulate the genes involved in oxidative metabolism have been recently identified [62-64]. ZNF224 is a multifunctional transcriptional repressor that interacts with different partners to modulate the expression of genes involved in controlling energy metabolism, cancer progression and apoptosis. ZNF224 was initially discovered for its involvement in the transcription of the aldolase A human gene [65]. ZNF236 is a glucose-dependent regulator and has been proposed to be a marker of diabetic nephropathy. Its transcript is upregulated in response to elevated levels of glucose. 

In addition to their role in regulating metabolic pathways, some KRAB-ZFPs appear to show intriguing functions that link metabolism and development. For example, the two KRAB-ZFPs Rsl1 and Rsl2 (regulator of sex-limitation) control the homeostasis of lipids and cholesterol, as well as modulating the expression of sexually dimorphic genes [66, 67]. Transcriptional control mediated by the Rsl genes was investigated by analyzing the liver transcriptome in wild-type, *rsl* knockout and transgenic mice overexpressing Rsl1 and Rsl2. About 7.5% of the 20000 transcripts assessed was responsive to Rsl and, within this group, male-predominant, female-predominant and not sexually dimorphic genes were collected. Genes involved in steroid/xenobiotic metabolism, immunity and metabolic balance were demontrated to be affected by Rsl regulation. Since these pathways are affected by the environment, it is likely that Rsl functions in the liver generate an adaptive response to external injuries (such as viral infection, exposure to toxins and changes in the composition of food). It is therefore possible that the expansion and diversification of the KRAB-ZFPs in humans caused an advantageous increase in the ability to adapt to environmental changes.

## KRAB ZINC FINGER PROTEINS AND DIFFERENTIATION

Expression studies suggested that KRAB-ZFPs, arisen from common ancestral genes, display individual patterns and, therefore, very specialized roles during the development and differentiation of the highest organisms. Although KRAB-ZFP genes that have been produced through multiple duplication events do not appear to be co-regulated, it is nonetheless intriguing to observe that several KRAB-ZFPs are involved in the modulation of cell proliferation and differentiation in haematopoiesis. 

The human ZNF268 has been shown to participate in the differentiation of erythroid cells. ZNF268 gene transcription is negatively modulated by GATA-1, the master regulator gene of haematopoiesis. The transcription factor GATA-1 binds to several erythroid-related genes regulating proliferation, differentiation and maturation of the erythroid-lineage cells. As a target of GATA-1, ZNF268 is repressed and, consequently, proliferation of K562 erythroleukaemia cells is induced and apoptosis and erythroid differentiation are inhibited [68]. Different alternatively spliced forms of ZNF268 have also been found and are aberrantly expressed in haematological malignancies [69]. These isoforms of ZNF268 might contribute to the onset of the diseases via corresponding protein products, which consequently have prognostic significance. 

The human ZNF300 has been linked to the role of the transcription factor PU.1 in regulating haematopoietic differentiation and leukaemia development [70]. In fact, ZNF300 gene transcription requires PU.1 gene activation in the APL-derived promyelocytes HL-60 cells. The PU.1 gene is a myeloid-specific transcription factor that binds to a purine-rich target sequence of several myeloid-specific gene promoters, thereby blocking myeloid haematopoiesis. The ZNF300 promoter contains a specific binding site for PU.1; its transcription depends on the presence of PU.1 in HL-60 but not Jurkat cells, thus indicating a cell-specific expression of ZNF300. Moreover, ZNF300 expression has been demonstrated to correlate with PU.1 expression during HL-60 cell differentiation. Finally, ZNF300 expression varies in different types of leukaemic blasts in the bone marrow samples of acute myeloid leukemia (AML) and chronic myeloid leukemia (CML) patients. Aberrant ZNF300 expression has also been observed in 5q-syndrome [71]. This is a specific subtype of primary myelodysplastic syndrome (MDS) that is due to a partial deletion of chromosome 5q13-q33. Altogether, these findings strongly suggest that ZNF300 has a crucial role during leukaemic blast differentiation through a PU.1-activated gene expression pathway. 

Several KRAB-ZFPs are differentially expressed during T-cell differentiation and activation. ZNF304 is a good example of this group of regulators that are, not by chance, clustered on chromosome 19. Basal transcription of ZNF304 mRNA in lymphocytes is induced following activation and, interestingly, its 6-hour peak correlates well with the peaks of IL-10, IL-4 and IFN-γ mRNA post-activation [72]. Although more results need to be accumulated about the role of ZNF304, the initial report demonstrated that ZNF304 was a new regulator of lymphocyte activation that also induced cytokines during lymphocyte differentiation.

Various KRAB-ZNFs have been found to play a fundamental role in the regulation of differentiation and morphogenesis. The ZNF230 gene, for example, encodes two different transcripts: the longer one of 4.4 kb is ubiquitously expressed, whereas the shorter form of 1 kb is detected only in the testis [73]. Interestingly, this latter form is absent in foetal testis and azoospermic patients, suggesting that ZNF230 might work in maintaning normal human male fertility. Another member of the KRAB-ZFP family, ZNF463, was found to be more expressed in normal fertile adults than in foetuses and azoospermic patients, also indicating a role of this protein in human spermatogenesis [74]. 

The expression of another KRAB-ZFP, AJ18, is modulated during differentiation in embryonic tibiae and calvariae, displaying very little expression in neonates and adults [75]. AJ18 gene transcription depends on Runx2, the well-known master gene for osteogenic differentiation. Overexpression of AJ18 in osteoblastic cells represses the markers of osteoblast differentiation [75]. 

NT2, a KRAB-ZFP that binds a specific sequence in the promoter of the gene encoding α2 collagen in mice, is involved in the regulation of cartilage construction [76]. NT2 expression is inversely correlated with *Col11*α*2*, suggesting that *Col11*α*2* promoter activity is inhibited by NT2 through binding to the 24-bp sequence via the KRAB domain. These results indicate that cartilage-specific expression of *Col11*α*2* is negatively regulated during embryonic development and chondrocyte differentiation.

ZNF359 and ZFP28, two evolutionarily related KRAB-containing zinc finger proteins, were identified from the human heart cDNA library [77]. Their expression is wide, but non-uniform in all the tissues of embryos and adults. Their specific expression in the heart suggests a potential function of these KRAB-ZFPs in differentiation and development in embryogenesis and cardiogenesis. 

The zinc finger protein 157 (zfp157) plays a role in alveologenesis, regulating the balance between luminal alveolar pStat5 and Gata-3 expressing cells in murine mammary glands [78].

## KRAB ZINC FINGER PROTEINS IN APOPTOSIS AND CANCER

Since the identity and the function of a cell is mainly determined by the activation or inactivation of its genes and as KRAB-ZFPs represent one of the largest families of transcriptional regulators in mammals, it is not surprising that these proteins are involved in controlling vital processes such as cell proliferation, apoptosis and neoplastic transformation [16]. The role of a KRAB-ZFP in these processes often appear to be dependent on the cellular context and the interaction with cell-specific molecular partners. For instance, besides its role in the transcriptional repression of genes involved in controlling energy metabolism [34, 62], ZNF224 has been shown to be an important regulator of apoptosis through the interaction with different molecular partners. Indeed, in the human erythroleukaemia cell line K562, ZNF224 displays a pro-apoptotic role by interacting with another zinc finger transcription factor, WT1. ZNF224 regulates WT1 transcriptional activity for several WT1 target genes, acting as a co-activator of WT1 in the regulation of proapoptotic genes and suppressing WT1-mediated transactivation of antiapoptotic genes [53, data not shown]. In bladder cancer cells, the interaction between ZNF224 and the cancer-testis antigen DEPDC1 plays a critical role in bladder carcinogenesis. In these cells, DEPDC1 acts as a transcriptional corepressor of ZNF224; the DEPDC1/ZNF224 complex represses the transcription of A20, a potent inhibitor of the NF-kB signalling pathway, thus resulting in the activation of the antiapoptotic pathway through NF-kB activation [79]. The overexpression of ZNF224, observed in breast cancer cells, sustains its role in cell transformation [80].

Another KRAB-ZFP, ZNF382, has been demonstrated to be involved in NF-kB signalling, but unlike ZNF224, it inhibits NF-kB. ZNF382 may also act by suppressing AP-1 signalling and repressing the expression of multiple oncogenes, thus exerting a pro-apoptotic role and inhibiting the proliferation of tumour cells. Epigenetic inactivation of ZNF382 through promoter CpG methylation has been observed in multiple carcinoma cell lines and also in common primary tumours. The attenuated expression of ZNF382 due to promoter CpG methylation activates NF-kB and AP-1 cancer signalling pathways during tumorigenesis [81]. 

Another KRAB-ZFP that is frequently methylated in cell lines and also in multiple primary tumours is ZNF545. Functional studies showed that ZNF545 is involved in the suppression of cancer cell growth by inhibiting ribosome biogenesis and inducing apoptosis [82]. These findings suggest that the epigenetic inactivation of KRAB-ZFPs that exhibit tumour-suppressive properties may be one of the molecular mechanisms involved in cancer development.The KRAB-ZFP ZBRK1 is a transcriptional repressor that was recently suggested to act as a tumour suppressor. ZBRK1 regulates the transcription of different genes involved in DNA damage response and in the cell cycle through the interaction with two different corepressors, BRCA1 and KAP1. In a BRCA1-dependent manner, ZBRK1 represses Gadd45 gene transcription [83], whereas p21 transcription is repressed by ZBRK1 through KAP1 recruitment [84]. Recently, ZBRK1 has been suggested to play a role in tumour angiogenesis, cooperating with CtIP/BRCA1 to repress angiopoietin-1 (ANG1) gene activation [85]. These data support a role of ZBRK1 as a potential tumour suppressor in mammary tumour cells. Recent findings suggest a role for ZBRK1 in cervical cancer, not only in suppressing tumour progression, but also in inhibiting metastasis by regulating metastatic genes. For example, it directly represses the transcription of the MMP9 metastatic gene [86].

Some members of the KRAB-ZFP family have been shown to be involved in p53 regulation via different molecular mechanisms. The complex p53 transactivation activity depends on the expression level of the p53 protein, its post-transcriptional modifications and its interaction with different molecular partners [87]. The KRAB-ZFP Apak (ATM and p53-associated KZNF protein) was recently identified as a specific inhibitor of p53-mediated apoptosis. It has been proposed that Apak modulates p53 activity by two different mechanisms. In the first, Apak interacts with p53 directly and recruits a protein complex containing the corepressor KAP1, the histone deacetylase HDAC1 and ATM to attenuate p53 acetylation, thereby repressing p53 activity. This results in the down-modulation of pro-apoptotic gene expression [88]. In the second mechanism, Apak competes with p53 to bind to the proapoptotic p53 target gene p53AIP1 to inhibit its expression [89]. ZNF307, another KRAB-ZFP involved in p53 regulation, reduces p53 protein levels. Downregulation of p53 protein levels by ZNF307 is probably due to the activation of MDM2 and EP300 gene expression, resulting in p53 degradation [90].

Besides that in cancer, some KRAB-ZFPs might play a central role in degenerative diseases. For example, ZNF746, a new parkin/interacting substrate also known as PARIS, leads to progressive loss of dopamine and DA neurons in the substantia nigra, a specific hallmark of Parkinson’s disease [91]. Genome-wide association (GWA) studies in Alzheimer's disease (AD), the most common cause of dementia, confirmed a putative involvement of ZNF224 in the onset of this degenerative disease. A SNP in the ZNF224 gene, indeed, was associated with both global AD neuropathology and global cognition [92]. Based on these preliminary results, targeted experiments are needed to clarify the role of the KRAB-ZFPs family proteins in the etiopathogenesis of these degenerative disorders.

## CONCLUSION

In this review article, we have reported the structural and functional features of some well-known KRAB-ZFPs (Table **1**). These proteins derived from ancestral genes through duplications and purifying selections during mammalian evolution, thus contributing to create novel functions. KRAB-ZFPs, belonging to the largest family of transcriptional repressors in eukaryotes, control the transcription of a large cohort of target genes through a common KRAB-dependent mechanism. In such way, they are involved in the regulation of crucial physiological and pathological processes as development, differentiation, metabolism, apoptosis and cancer. Although questions related to the vertebrate-specific gene expression and the cellular functions remain to be elucidate, isolation and characterization of new members of the KRAB-ZFPs family could improve a more complete understanding of the biological roles of this protein family.

## Figures and Tables

**Table 1.** Structural and Functional Features of Some KRAB-ZNFs

Metabolism							
Protein	Species	Chromosomal Localization	KRAB	Zinc Fingers	Expression Profile	Functional Role	References
ZNF202	Human	11q.23.3	KRAB+SCAN	8	ubiquitous	Lipids Metabolism	[56-58]
ZNF224	Human	19q13.2	KRAB-A+b box	19	ubiquitous	Glycolysis Oxidative metabolism	[62, 63, 65]
ZNF236	Human	18q22.3-23	KRAB-A box	25/30	ubiquitous	Glucose metabolism	[64]
Rsl1	Mouse	13B3	KRAB-A+B box	3	liver	Lipids homeostasis Sexual dimorphism	[66, 67]
Rsl2 (Zfp429)	Mouse	13B3	KRAB-A+B box	3	liver	Lipids homeostasis	[66, 67]
** Development and Differentiation**							
**Protein**	**Species**	**Chromosomal Localization**	**KRAB**	**Zinc Fingers**	**Expression Profile**	**Functional Role**	**References**
ZNF268	Human	12q24.33	KRAB-A+B box	14	erythrocytes	haematopoiesis	[68, 69]
ZNF300	Human	5q33.1	KRAB-A+b box	12	promyelocytes	haematopoiesis	[70, 71]
ZNF304	Human	19q13.4	KRAB-A box	13	lymphocytes	lymphocyte activation	[72]
ZNF230	Human	19q13.31	KRAB-A box	4	testis	spermatogenesis	[73]
ZNF463	Human	19q13.42	KRAB-A+B box	12	testis	spermatogenesis	[74]
AJ18	Rat	11B1.3	KRAB-A box	11	bone	osteogenesis	[75]
NT2	Mouse	16A1	KRAB-A box	9	cartilage	development	[76]
ZNF359	Human	16q22	KRAB-A+B box	16	heart	cardiogenesis	[77]
ZFP28	Human	19q13.41	KRAB-A box	14	heart	cardiogenesis	[77]
ZFP57	Human	6p22.1	KRAB-A box	6	ovary, testis	imprinting	[40, 41]
Zfp157	Mouse	5G2	KRAB-A+B box	11	mammary gland	alveologenesis	[78]
** Apoptosis and Cancer**							
**Protein**	**Species**	**Chromosomal Localization**	**KRAB**	**Zinc Fingers**	**Expression Profile**	**Functional Role**	**References**
ZNF224	Human	19q13.2	KRAB-A+b box	19	ubiquitous	Control of apoptosis	[53, 79, 80]
ZNF382	Human	19q13.12	KRAB-A+B box	6	heart	Tumor suppressor gene	[81]
ZNF545	Human	19q13.12	KRAB-A box	8	ubiquitous	Tumor suppressor gene	[82]
ZBRK1	Human	19q13.41	KRAB-A box	8	skeletal muscle	Tumor suppressor gene	[83-86]
Apak	Human	19q13.12	KRAB-A+b box	19	ubiquitous	Regulator of apoptosis	[88, 89]
ZNF307	Human	6p21	KRAB+SCAN	7	ubiquitous	p53 degradation, apoptosis	[90]
** Degenerative neurological diseases**							
**Protein**	**Species**	**Chromosomal Localization**	**KRAB**	**Zinc Fingers**	**Expression Profile**	**Functional Role**	**References**
ZNF746	Human	7q36.1	KRAB-A box	4	ubiquitous	Parkinson’s disease	[91]
ZNF224	Human	19q13.2	KRAB-A+b box	19	ubiquitous	Alzheimer’s disease	[92]
